# Veterinary Department, Detroit College of Medicine

**Published:** 1895-09

**Authors:** 


### VETERINARY DEPARTMENT, DETROIT COLLEGE OF MEDICINE.

This school announces on the cover-page of its catalogue that the course this year will last seven months, while on page 8 they state that the session shall continue six months. L. E. Schell, M.D., professor of animal physiology, has been succeeded by S. S. Shields, M.D. M. L. High, M.D., V.S., has been added to the teaching staff as professor of materia medica and therapeutics. A course on horseshoeing has been added, to last three months. No changes are noted in the matriculation examination or requirements for graduation.

